# Dietary Melatonin Boosts Reproduction and Growth Performance of Ornamental Fish Giant Danio (*Devario aequipinnatus*): A Transformative Approach for Scrapping Wild-Caught Fish Business

**DOI:** 10.1155/anu/5540109

**Published:** 2025-02-13

**Authors:** Khusbu Samal, Pradyut Biswas, Soibam Khogen Singh, Pronob Das, Reshmi Debbarma, Suparna Deb, Dharmendra Kumar Meena, Simanku Borah

**Affiliations:** ^1^Department of Aquaculture, College of Fisheries, Central Agricultural University, Lembucherra 799210, Tripura West, India; ^2^Krishi Vigyan Kendra, ICAR Research Complex NEH Region, Manipur Centre, Ukhrul 795142, India; ^3^ICAR-Central Inland Fisheries Research Institute, Regional Centre, Guwahati 781006, Assam, India; ^4^Open Water Aquaculture Production and Management Division, ICAR-Central Inland Fisheries Research Institute, Barrackpore 700120, West Bengal, India

**Keywords:** *Devario aequipinnatus*, gonadosomatic index, melatonin, ovarian, physiological

## Abstract

The present global trade of endemic ornamental fishes is heavily relied wild-caught species that concerns long-term sustainability. This study examined the effects of dietary melatonin on the reproductive performance and health of *Devario aequipinnatus* (giant danio). A basal diet of 35% protein (basal diet as control) was supplemented with four different doses of melatonin (2 mg [M1], 10 mg [M2], 50 mg [M3] and 100 mg [M4] per 100 g of feed) given to experimental groups in triplicate. Fish (average weight: 1.13 ± 0.15 g) was stocked in tanks (*n* = 10) and fed 5% of body weight twice daily. After 60-day feeding, key reproductive metrics such as the gonadosomatic index (GSI), fecundity, egg diameter and histological changes were analysed along with growth and physiological status. The GSI was observed to be better with the increase in dosage and was higher in M3 (3.05 ± 0.03%) (*p*  < 0.05). Histological examination revealed the presence of advanced oocyte stages IV and V in M3, while higher (>50 mg) melatonin levels suppressed the GSI. Egg diameter increased with the dosage of melatonin up to 50 mg/100 g (1.18 ± 0.6 mm). Testicular development was most advanced in 50 mg (M3) dose of melatonin with significant higher appearance of stage II spermatids or spermatozoa. In addition, M3 exhibited markedly elevated levels of vitellogenin (VG) (3.38 ± 0.22 nmol/L) in female fish and testosterone (16.4 ± 1.11 nmol/L) in male fish compared to the control. Broken-line regression analysis indicates that the optimal dose for improved growth performance was identified at 63 mg/100 g of diet. Melatonin supplementation significantly increased (*p*  < 0.05) haematological indices such as haematocrit value, leucocyte count, haemoglobin (Hb) and packed cell volume (PCV) compared to the control, except for the 100 mg group (M4). Although stress markers such as glucose and cortisol were similar to the control, there was a plausible rise in the amount of antioxidant enzyme (*p*  < 0.05) in the melatonin groups. Overall findings of the study demonstrate the potential of melatonin improving the reproductive and physiological status of endemic ornamental fish for accelerating the captive breeding programme for sustainable trade.

## 1. Introduction

Aquaristics or ornamental fish culture is one of the most popular hobbies in the world, second only to photography, with millions of enthusiasts worldwide [1]. According to the Observatory of Economic Complexity (OEC), the global trade value of ornamental fish reached $339 million in 2022, representing ~0.0014% of the total global trade value [2]. The growing demand for ornamental fish, combined with the need for responsible management of wild stocks, highlights the importance of standardizing breeding protocols for popular species [3]. Research shows that fish can be artificially induced to spawn by manipulating their hormonal axis [4]. Significant advancements have also been made in enhancing the breeding performance of commercial fish by adjusting water temperature and photoperiod, which stimulate hormonal activity [5]. These factors primarily influence the release of reproductive hormones, regulated by melatonin produced by the pineal gland and retina. Inducing early maturation in fish through the administration of exogenous hormones offers a promising approach to achieving artificial breeding in captivity, ensuring a consistent supply of ornamental fish [6].

The use of dietary melatonin in ornamental aquaculture represents a transformative approach to reducing reliance on wild-caught fish, offering a sustainable alternative for the industry. Wild capture of fish has long been associated with overexploitation of natural populations, ecological imbalances and habitat degradation. By enhancing reproduction, growth and overall health in species like the giant danio (*Devario aequipinnatus*), melatonin-enriched diets provide a viable method for consistent and efficient breeding in controlled environments. This not only ensures a steady supply of high-quality ornamental fish but also mitigates pressure on wild stocks, paving the way for a more ethical and environmentally responsible aquaculture model. Such advancements mark a new paradigm in the ornamental fish trade, fostering sustainability while meeting the growing market demand.

Melatonin (N-acetyl-5-methoxytryptamine) is a pineal gland hormone that acts as a strong regulator of the circadian and reproductive cycles in seasonal breeders such as fish [5]. *D. aequipinnatus* is an ornamental fish and is quite sensitive to the environmental changes which is definitely the main reason for discussing about its better seed quality, breeding and gamete quality. Melatonin hormone secretion is related to photoperiod and influences animal circadian rhythms [4, 7]. Previous research suggests that melatonin may play a role in regulating maturation and spawning via the hypothalamo–pituitary–gonadal (HPG) axis [4]. Melatonin travels directly to the pituitary gland via pituitary melatonin receptors, where it activates gonadotropin-releasing hormones (GnRHs). Previous research found that exogenous melatonin had a dose-dependent effect on the reproductive performance of Nile tilapia, *Oreochromis niloticus* [8]. Furthermore, there is evidence that exogenous melatonin reduces oxidative stress in fish by stimulating antioxidative enzymes [9], which is primarily due to its lipophilic nature. Melatonin improves oocyte quality by reducing oxidative stress, according to Tamura et al. [10]. Melatonin, in particular, can act as an antioxidant on many cells in the ovarian follicles, activating key enzymes such as superoxide dismutase (SOD), catalase (CAT) and glutathione peroxidase (GPx), which can metabolize free radicals and reduce oxidative stress [10]. Furthermore, haematological parameters can be used as useful indicators to support the role of exogenous melatonin on fish health [11].

Among several indigenous ornamental species, *D. aequipinnatus* (common name: giant danio), a vibrant and brilliantly striped hill stream fish of the Cyprinidae family, is in high demand on the global market (priced at 1.2 US dollar/piece). It is found in schools at the surface of small high-gradient upland streams in shaded, mid-hill clear waters with pebble or gravel substrates. Because of the high demand in the international market, there has been indiscriminate harvesting of fish from wild resources. In this study, an attempt was made to restructure the ornamental trade of this species with captive maturation using a dietary approach. Previous success on the potential influence of exogenous melatonin on other food fishes has been replicated in this study on ornamental fishes. The objective of this study was to determine the effect of dietary melatonin on *D. aequipinnatus* growth, reproductive performance (as measured by changes in hormonal levels, key indices and tissue level change), oxidative stress and overall health. The findings of this study will assist us in comprehending better the role of melatonin and how it can be used to improve the reproductive success of this species and help us to find a better way for quality seeds.

## 2. Materials and Methods

### 2.1. Animal Ethics

Experimental fishes were handled and raised according to Indian laws. The study protocol and experimental endpoints follow the CPCSEA's guidelines on animal care and use in scientific research. The Institutional Ethics Committee (IAEC) of the College of Fisheries, Central Agricultural University (I), Tripura, India, approved the study (Approval Letter No. CAU-CF/48/IAEC/2018/01, 08/04/2024).

### 2.2. Experimental Animals

Fishes were cast netted from Khowai River, Tripura, India, and acclimatized in aquariums for 15 days before the experiment. After having arrived at the aquarium house, injured fish were separated, and the stock was disinfected with 10 ppm acriflavine to prevent pathogenic infection. During acclimatization, fish were fed commercial diets, and water was appropriately aerated using a centralized blower and supplied using stone aerators to the individual tanks.

### 2.3. Experimental Feed Preparation

Melatonin (HiMedia) as a chemical is available in a powdered form. Four experimental diets were formulated (Pearson's method) by incorporating four different doses of exogenous melatonin at inclusion levels of 2 mg (M1), 10 mg (M2), 50 mg (M3) and 100 mg (M4) per 100 g of feed into a base diet formulated using the sources listed in Table 1. The prepared basal diet contained 35% protein and was used as the control diet. The level of incorporation was determined based on our previous experiment with related species (data unpublished). The powdered form of melatonin was dissolved in absolute alcohol [12]. The solution was then added to the feed and thoroughly mixed to ensure uniform distribution. Following this, the feed box's lid was left off overnight to allow the alcohol to evaporate. The prepared feed was subsequently wrapped in aluminium foil and stored in a dark place.

### 2.4. Experimental Design and Set-Up

The research was conducted in 40-L aquariums housed in the Wet Laboratory of the College of Fisheries, Central Agricultural University, India. The source water was borewell water that was aerated for 4 days in a 1000 L storage tank. There are 15 aquariums divided into 4 treatments and 1 control, where 3 aquariums were considered under each treatment. Equal ratio of male and females was stocked as per the morphology. The design of the experiment was completely randomized (CRD). After filling the tanks with seasoned water to a volume of 30 L, 10 fish (*n* = 10) from the acclimatization tank were transferred to each tank. On the day of stocking, feeding was stopped. All aquariums were equipped with a central blower that provided continuous aeration. The aquarium was set to a standard photoperiodic regime of 12:12 light/dark.

### 2.5. Feeding of Animals

Fish were fed with the designated experimental diets. Initially, 5% of body weight was fed twice daily at 09:00 and 16:00 h. Based on the fish biomass, the amount of feed was adjusted every 2 weeks. The fish were fed the melatonin incorporated diet twice daily by hand feeding. Uneaten feed and excreta was removed from the system after the subsequent hour of feeding by siphoning.

### 2.6. Water Quality Analysis

The key water quality parameters like pH, temperature, dissolved oxygen (DO), hardness and alkalinity were examined on fortnightly basis and are presented in Table 2. The standard method for estimation of water quality parameters described by APHA [13] was followed in this study.

### 2.7. Blood Collection and Tissue Sample Preparation

After the 60 days of feeding with the melatonin-incorporated diet, fishes were anaesthetized using clove oil (50 μg L^− 1^). The harvested fishes were left in the euthanized solution for 10 min or until cessation of the opercular activity. The blood collection of the sampled fishes was done immediately. For analysis of the different immuno-biochemical parameters, six fishes (*n* = 6) from each treatment three male and three female were collected and used. The liver, gill and muscle of the fish were aseptically removed by dissection on a cold plate. Five per cent tissue homogenate was prepared by using chilled sucrose solution (0.25 M) and then centrifuged at 5000 rpm for 15 min and stored at −20°C for further analysis. The same fishes were previously used for blood collection by caudal vein puncture. Aliquot of the blood was collected with 10% EDTA coated tubes. For plasma, blood was drawn into centrifuge tubes without EDTA and then centrifuged at 3000 rpm for 10–15 min. Thereafter, the supernatant plasma was pooled and kept in a deep refrigerator (−20°C), until further use. For analysis of gene expression, the samples of the brain, liver and gonads were collected and preserved in TRIzol in −20° C till its maceration and further process done.

### 2.8. Growth Parameters

Growth sampling was performed on fortnightly basis to monitor the growth and accordingly adjust the feeding ration. The following parameters were calculated at the end of the experiment using the following formula:

  $$\begin{array}{c} \text{Body weight gain }\left(\%\right)=\text{final weight (g) − initial weight (g) }\times \text{ 100 initial weight (g)}, \end{array}$$

  $$\begin{array}{c} \text{Specific growth rate (SGR) }\left(\%/\text{day}\right)=\frac{\ln\;\left[\left(\text{final weight (g)}\right.-\ln\;\left(\text{initial weight (g)}\right.\right]}{\text{Experimental period in days}}\times 100\;. \end{array}$$

### 2.9. Reproductive Performance

The reproductive performance of experimental animals was estimated in terms of reproductive parameters like fecundity [14] and gonadosomatic index (GSI). The GSI was measured in 30 days and 60 days. For initial GSI, one female fish from each treatment was selected. Egg diameter was measured following the method of Yaron [15]. The following formula was used:

  $$\begin{array}{c} \text{Total fecundity}=\frac{\left(\text{No. of eggs in the sample }\times \text{ weight of the gonad}\right)}{\text{Weight of the sample}}, \end{array}$$

  $$\begin{array}{c} \text{Gonadosomatic index (GSI)}=\frac{\text{ Weight of gonad (g)}}{\text{ Bodyweight of fish (g)}}\times 100. \end{array}$$

For sperm parameters, males were squeezed, as described above, and sperm from several males was pooled to produce a final volume of at least 8 mL/trial. This sample was diluted to 16,109 cells/mL as measured on a haemocytometer, and then portions diluted with HBSS to yield 10^8^, 10^7^, 10^6^, 10^5^ and 10^4^ cells/mL. the sperms are collected quite a less amount from the fishes per replicates due to the small size of the fishes.

### 2.10. Reproductive Hormone

The testosterone level was checked using commercial ELISA kit (Bioassay Technology Laboratory, EA0009Ge). The samples were obtained from all the treatments when dissected the testes part of the male fishes from the replicates and mixed to form a homogenate and centrifuged it. Supernatant of the samples were then added to the precoated plate. The female testosterone was investigated from the muscle and liver tissues of females. The basis of this assay was that the colour intensity developed is inversely proportional to the testosterone concentration in the sample. The concentration of testosterone in the sample is then determined by comparing the optical density of the samples to the standard curve. The vitellogenin (VG) kit (Fish Vitellogenin ELISA Kit MBS010726) works with Quantitative Sandwich ELISA which was for lab reagent/research use only. This kit was intended to be used to determine the level of VG in undiluted original muscle tissue homogenates samples. The reading for the hormone analysis the absorbance was 450 nm.

### 2.11. Gonadal Histology

Histological analysis was performed according to protocol described by Roberts and Ekman [16]. Gonadal sections of the fish were collected at the end of the trial for histological analysis. For histology, two fishes, one male and one female, were collected from each treatment, and the sections were collected aseptically and were fixed in Bouin's fixative (glacial acetic acid 5%, formaldehyde 9% and picric acid 0.9%) in ration of 1:10 w/v for 48 h. Tissue processing was done in automatic tissue processor (Thermo Scientific, Shandon Citadel 2000), followed by paraffin block preparation in Histocenter (Thermo Scientific, Shandon HistoCentre). Sectioning of paraffin embedded was done using Rotary Microtome (Leica, RM2245). Sections were cut in 5–6 μm thickness, which follows staining with haematoxylin and eosin (H&E) (HiMedia) and then mounted with DPX. Slides were observed and examined under Lieca DM 750 microscope at 10× magnification.

### 2.12. Haematological Parameters

Total erythrocyte count (TEC), total leukocyte count (TLC) and packed cell volume (PCV) were calculated following method described by Schaperclaus [17]. Total haemoglobin (Hb) content was estimated using a Sahli Haemometer (Marinefield, Germany), following the method of Schaperclaus [17]. Reading of the Hb content was determined from the scale given in the measuring tube.

### 2.13. Antioxidant Enzyme Assay

CAT assay was done as per Takahara [18]. Fifty microlitres of the tissue homogenate was added to 2.45 mL of phosphate buffer (50 mM, pH 7.0), and the reaction was initiated by adding 1.0 mL of H_2_O_2_ solution. The absorbance was measured at 240 nm at 30-s intervals for 2 min. The enzyme activity was expressed as nmol of H_2_O_2_ decomposed/min/mg protein. The SOD activity enzyme was assayed according to the method of Misra and Fridovich [19] which is based on the oxidation of epinephrine–adrenochrome transition by the enzyme. One unit of SOD activity was the amount of protein required to give 50% inhibition of epinephrine auto oxidation. Malondialdehyde (MDA) activity was assayed by Hodges et al. [20] method with a slight modification done by Turan and Tripathy [21]. Twenty per cent w/v trichloroacetic acid (TCA) without TBA and 20% w/v TCA containing 0.25% TBA were used. Absorbance of supernatant was recorded at 440, 532 and 600 nm, and MDA content was calculated as described by Turan and Tripathy [21] as an index of lipid peroxidation.

### 2.14. Stress Parameters

The cortisol level in plasma was determined using an ELISA-based cortisol test kit (Cal biotech, Inc., catalogue no. CO368S). Upon the addition of the substrate, the intensity of colour is inversely proportional to the concentration of cortisol in the samples. A standard curve was prepared relating colour intensity to the concentration of the cortisol. The final reading was taken with the absorbance of 450 nm. The determination of glucose was performed using a diagnostic kit (Coral Clinical Systems, India) that is based on the GOD/POD method [22].

### 2.15. Statistical Analysis

Initially, data were subjected to Shapiro–Wilk and Levene's test for addressing the normality and homogeneity of variances. All the data were subjected to one-way analysis of variance (ANOVA) using SPSS version 25.0 software for Windows. Tukey's multiple comparisons post hoc test was chosen for comparison between the treatments, and a significance level of 5% was adopted. The optimum concentration of melatonin was ascertained using a broken line regression model. All data were expressed as mean ± standard error (SE).

## 3. Results

### 3.1. Effect on Reproductive Maturation

**Figure 1:**
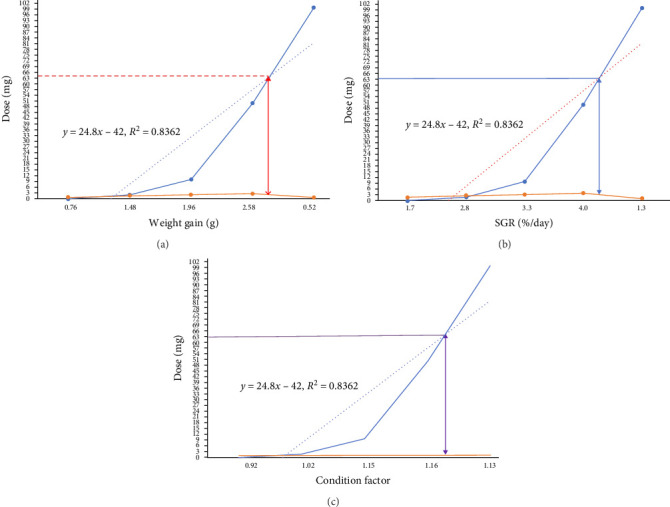
Relationship between the (a) weight gain (WG, g), (b) specific growth rate (SGR) (%/day) and (c) condition factor (CF) and the dietary melatonin level based on two-slope broken-line regression analysis.

Effects of dietary melatonin on reproductive scores are presented in Figure 1. Fecundity and egg diameter of *D. aequipinnatus* were observed to be increased with increase in dietary melatonin up to a level of 50 mg 100 g^−1^. Beyond this concentration, no significant improvement of fecundity and egg diameter was observed. With regard to GSI, melatonin supplementation beyond 2 mg 100 g^−1^ significantly increases GSI with increase in concentrations for the initial 30 days. However, at 60^th^ days, the higher GSI was observed in M3 (50 mg 100 g^−1^) followed by M4, M2, M1 and control.

### 3.2. Histological Changes in Gonads

**Figure 2:**
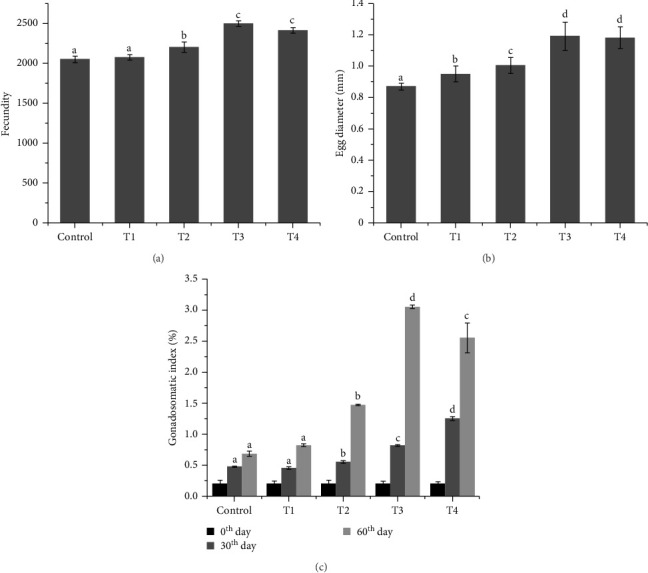
The calculated reproductive scores: (a) fecundity, (b) egg diameter and (c) gonadosomatic index (GSI) values of *Devario aequipinnatus* fed different experimental diets at different sampling points. Values are presented as means ± SE of three replicates. Different superscript letters (a, b, c, d) in each row show significant differences among treatments by Tukey's test (*p*  < 0.05).

The progression and acceleration of ovarian maturity in *D. aequipinnatus* subjected to different levels of dietary melatonin are depicted in Figure 2. The study revealed that the M3 supplemented group, which received 50 mg melatonin per 100 g of feed, had the higher percentage of matured oocytes at stages IV and V. These included vitellogenic oocytes (stage V) and alveolar oocytes (stage IV), which were clearly visible. In the M4 supplemented group, which was 100 mg melatonin per 100 g of feed, there was a noticeable increase in the number of atretic oocytes compared to other treatments. The M1 (2 mg 100 g^−1^ of feed) and M2 (10 mg 100 g^−1^ of feed) supplemented groups primarily showed early-stages of oocytes by the end of 60 days. There were no significant changes (*p*  > 0.05) in ovarian maturity between these two groups.

**Figure 3:**
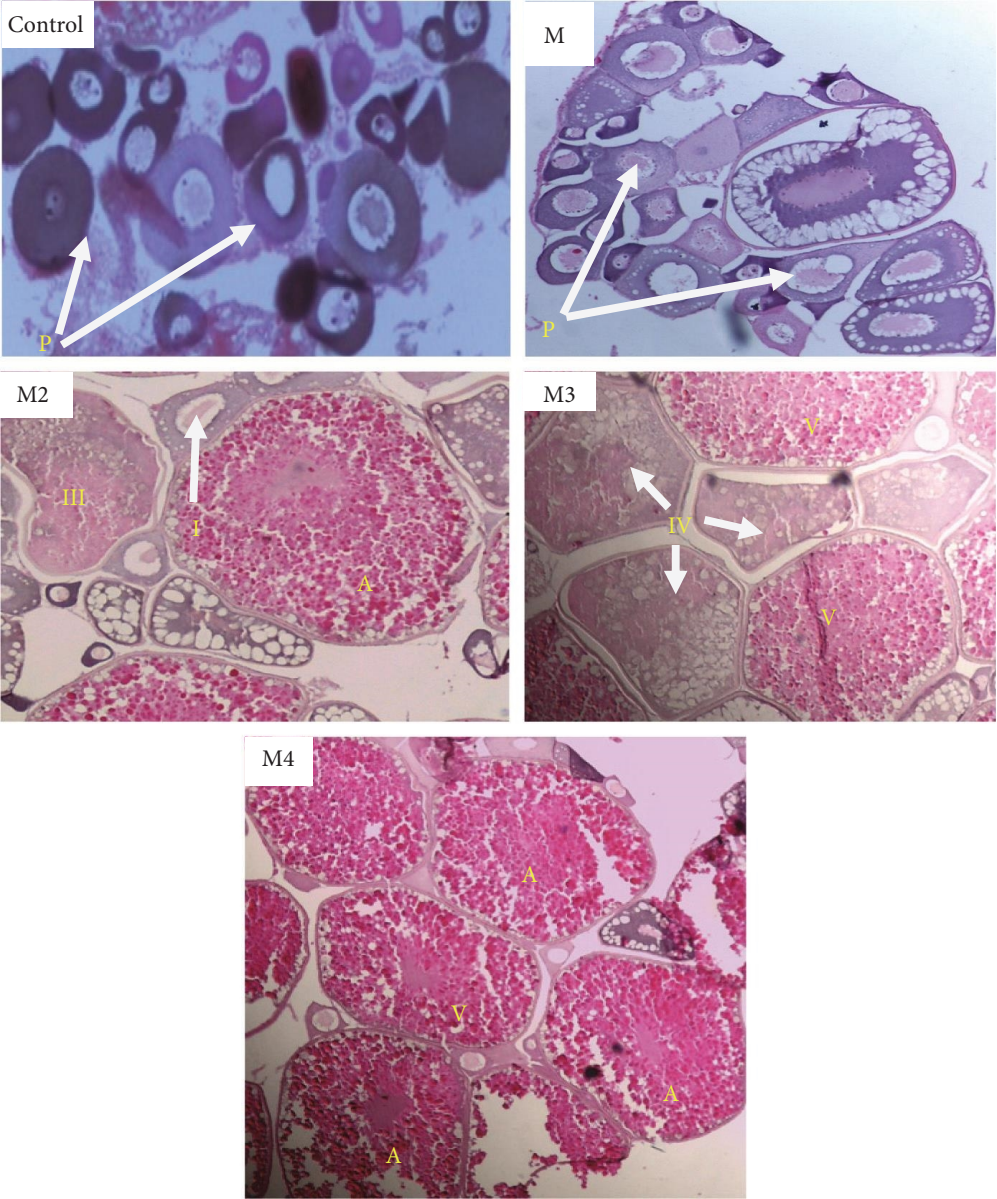
Histomicrographs showing the progression of ovarian maturity in different groups with varying melatonin levels in the diets of *Devario aequipinnatus* during 60-day feeding. A, atretic oocyte; I, stage I oocyte; II, stage II oocyte; III, stage III oocyte; IV, stage IV oocyte; P, perinuclear oocytes; V, stage V oocyte.

The progression of testicular maturity in male *D. aequipinnatus*, as influenced by dietary melatonin levels, is shown in Figure 3. Histological examination of the male gonads observed that the M3 dose of melatonin (50 mg melatonin per 100 g of feed) had a significantly higher presence of stage II spermatids or spermatozoa. The control (0 mg) and M1 doses (2 mg melatonin per 100 g of feed) exhibited a higher abundance of spermatogonia and spermatocytes, that is, primary and secondary spermatocytes. In the M4 group (100 mg 100 g^−1^ of feed), less spermatid appearance was noted as compared to M3 as well as other doses

The results for the effect of melatonin on gonads clearly described that under a trial of 60 days, the effect of melatonin showed a significant improvement in the gonads that would have possibly accelerated the process of reproduction in a dose-dependent manner. In the study, the M4 dose-oriented fish had a suppressive effect in both ovaries and testes which directed us to the optimal dose of melatonin as 50 mg 100 g^−1^ (M3) diet showing the noticeable positive effects on the fish.

### 3.3. Sperm Quantity and Quality

**Figure 4:**
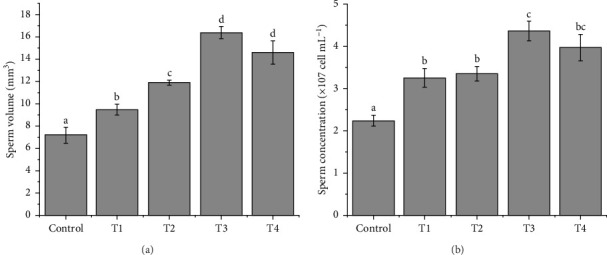
The calculated sperm scores: (a) volume and (b) concentration of *Devario aequipinnatus* fed different experimental diets. Values are presented as means ± SE of three replicates. Different superscript letters (a, b, c, d) in each row show significant differences among treatments by Tukey's test (*p*  < 0.05).

Melatonin-treated groups showed a plausible increase in volume and concentration of sperms when compared to control (*p*  < 0.05) as noted in Figure 4. Among the treated groups, M3 (50 mg 100 g^−1^) dose fish had the highest sperm volume and concentration 16.4 ± 0.54 µm^3^ 4.36 ± 0.23 × 10^7^ cell mL^−1^, respectively. Further, melatonin level exceeding 50 mg 100 g^−1^ did not showed any significant improvement, that is, M4 (100 mg 100 g^−1^).

### 3.4. Levels of Sex Hormones

Table 3 outlines the level of reproductive hormones in male and female fish in this study. In male fish, the higher level of testosterone (16.4 ± 1.11 nmol^−1^) was recorded in the M3 dose group (50 mg 100 g^−1^), whereas the control group recorded the lowest value (4.37 ± 0.39) (*p*  < 0.05). In female fish, very little level of testosterone was detected, and there was no statistically significant difference (*p*  > 0.05) among the treatments and control dose groups of melatonin supplementation. The variation of VG levels were detected from the liver and muscle of the female fish. The results showed that VG levels significantly increased with increasing melatonin supplementation (*p*  < 0.05) in both the liver and muscle. The highest levels were exhibited in M3 with measurements of 3.38 ± 0.22 nmol^−1^ and 1.83 ± 0.03 nmol^−1^ in the liver and muscle, respectively.

### 3.5. Effects on Fish Growth

Effect of melatonin supplementation on growth rate of *D. aequipinnatus* is depicted in Table 4. The result showed that the growth rate of the fish was significantly affected by the amount of melatonin in their diet (*p*  < 0.05). The higher MBWG was recorded as 2.58 0.18 g in M3 (50 mg 100 g^−1^) groups (*p*  < 0.05), while the control and M1 group had lowest MBWGs of only 0.52 0.05 g and 0.76 0.05 g, respectively. Specific growth rate (SGR) was observed to be significantly higher in M3 (3.95 0.16) and M (3.32 ± 0.28) groups as compared to other treatments (*p*  < 0.05). At a supplementation level of 50 mg 100 g^−1^ (M3), melatonin's growth-promoting action in fishes becomes apparent. According to the broken-line regression of weight gain (WG), SGR and condition factor (CF), the optimum level in *D. aequipinnatus* was found to be 63 mg of melatonin in 100 g of the prepared diet (Figures 1–3).

### 3.6. Immune and Antioxidant Enzyme Status

The results of the immune and antioxidants enzyme status (RBC, WBC, Hb and PCV) were shown in Tables 5 and 6. The TEC and TLC were recorded to be significantly higher (*p*  < 0.05) in M3 dose of melatonin (5.48 ± 0.13; 63.34 ± 0.01), while M4 showed a decrement in the level compared to control. The PCV was also higher in M4 (57.14 ± 1.34), while no significant change in levels was noticed between control, M1 and M2 dose of melatonin. The fish fed with M4 dosage of melatonin had the lowest level of TEC, TLC and PCV. Except for the M4 dose of fish, which showed elevated levels compared to the control, there was no statistically significant difference in Hb levels (*p*  < 0.05) across all melatonin-supplemented treatments. The M4 group of fishes (100 mg 100 g^−1^), however, showed decreased levels of blood parameters compared to the other dosage of melatonin treatments. The results of immune and antioxidant enzyme status, including RBC, WBC, Hb and PCV levels, are presented in Tables 5 and 6. TEC and TLC were significantly higher (*p*  < 0.05) in the M3 melatonin dose group (5.48 ± 0.13 and 63.34 ± 0.01 million cells mm^−1^, respectively). In contrast, the M4 group showed a reduction in these parameters compared to the control group. PCV was the highest in the M3 group (57.14 ± 1.34), while no significant changes were observed between the control, M1 and M2 groups. Fish in the M4 group exhibited the lowest TEC, TLC and PCV levels. Hb levels did not show any statistically significant differences across the melatonin-supplemented groups, except in the M4 group, which had elevated Hb levels compared to the control. However, the M4 group (100 mg 100 g^−1^) showed decreased levels of blood parameters compared to other melatonin-treated groups. Compared to the control group, all melatonin groups showed significant increase in SOD and CAT levels, along with a decrease in MDA levels (*p*  < 0.05). Among the melatonin-treated groups, the M3 group (50 mg 100 g^−1^) exhibited the highest levels of SOD and CAT and the lowest levels of MDA.

### 3.7. Effects on Glucose and Cortisol Level

**Figure 5:**
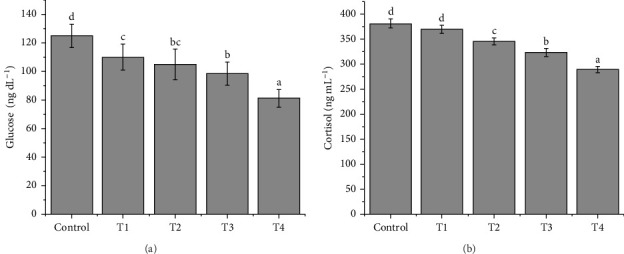
(a–b) The calculated stress scores: (a) glucose and (b) cortisol levels of *Devario aequipinnatus* fed different experimental diets. Values are presented as means ± SE of three replicates. Different superscript letters (a, b, c, d) in each row show significant differences among treatments by Tukey's test (*p*  < 0.05).

Figure 5 demonstrated that the supplemented groups had a considerably reduced level of the hormones “Glucose” and “Cortisol” in comparison to the control group. Fish fed on 50 mg 100 g^−1^ melatonin-supplemented diet had the lowest cortisol levels (323 ± 8.33 ng mL^−1^) compared to any of the groups. The lowest glucose level of 98.5 ± 2.21 ng mL^−1^ was also found in the same group as the lowest cortisol level showing lower stress.

## 4. Discussion

The present study demonstrated that the supplementation of melatonin to the diet benefited to the healthy maturation of *D. aequipinnatus* in captivity. Our specific objective in this work was to investigate how melatonin affects the early maturation of the native ornamental fish species, *D. aequipinnatus*, by measuring fish growth, observing different gonadal developmental stages histologically, examining the quality of gametes, measuring levels of sex hormone and examining its relationship to serum antioxidant status and stress level at different doses. The results of this study will help us to comprehend how melatonin affects fish development and reproduction.

### 4.1. Effect on Reproductive Maturation

In this study, melatonin supplementation demonstrated positive effects on the reproductive indices of fish. The melatonin-treated groups exhibited higher values for key reproductive parameters, including egg diameter, fecundity and GSI (Figure 2). Melatonin is known to influence the seasonal reproductive cycle by accelerating gonadal maturation through its action on the brain–pituitary–gonadal axis. Research indicates that exogenous melatonin can have either positive or negative effects on gonadal function, depending on the species and reproductive status of the fish [23, 24]. These effects are highly species-specific. Our findings align with previous studies that reported increased fecundity due to exogenous melatonin in species such as zebrafish (*Danio rerio*) [25, 26], *Channa punctatus* [27, 28] and *Clarias macrocephalus* [29, 30]. However, in other species like *Oryzias latipes* [31, 32] and *Heteropneustes fossilis* [24, 33], melatonin has been documented to exert antigonadal effects. The impact of melatonin varies depending on the species, the stage of gonadal development and the dosage administered [26, 34]. In our study, a supplementation dose of 50 mg 100 g^−1^ was the most effective treatment, significantly accelerating gonadal maturation. This dose-dependent effect was reflected in the higher GSI, fecundity and egg diameter observed in the M3 group, identifying it as the optimal supplementation level for this species (Figure 2).

### 4.2. Histological Changes in the Ovary

The annual reproductive cycle of fish gonads comprises four distinct phases: the preparatory phase, characterized by mostly oogonia and a few stage I oocytes; the prespawning phase, featuring stage II oocytes; the spawning phase, marked by yolk-filled stage III oocytes; and the postspawning phase, where many atretic or postovulatory follicles are present in female fish, as described by Maitra et al. [24, 28]. The development of ovarian and testicular maturity in *D. aequipinnatus* fed different levels of dietary melatonin is illustrated in Figures 4 and 5. Fish in the T3 group (50 mg/100 g feed) exhibited the highest percentage of mature oocytes (stages IV and V). Specifically, vitellogenic oocytes (stage V) and alveolar oocytes (stage IV) were prominent in this group. Wallace and Selman [30, 35] highlighted the melatonin's role as a key regulator of vitellogenesis—a process where the liver synthesizes VG, the precursor protein for yolks, under oestrogen influence. However, Maitra et al. [24, 32] reported that exogenous melatonin accelerates the transition from stage I to stage II oocytes during the preparatory phase but slows down the progression during the prespawning and spawning phases. In our study, the T4 group (100 mg/100 g feed) showed a higher number of atretic oocytes compared to other treatments, indicating that doses exceeding 50 mg/100 g may have a restrictive effect on gonadal development. Melatonin also directly impacts follicles by enhancing germinal vesicle breakdown (GVBD), as noted in previous research on zebrafish (*D. rerio*) and *Catla catla* [31, 33, 36]. Interestingly, studies suggest that the stimulatory effects of melatonin and maturation-inducing hormone (MIH) on GVBD are diminished when oocytes are incubated with serotonin (a melatonin precursor), melatonin or both [24, 34]. This interplay between serotonin and melatonin could provide further clarity to the results observed in our trial.

### 4.3. Histological Changes in the Testes

**Figure 6:**
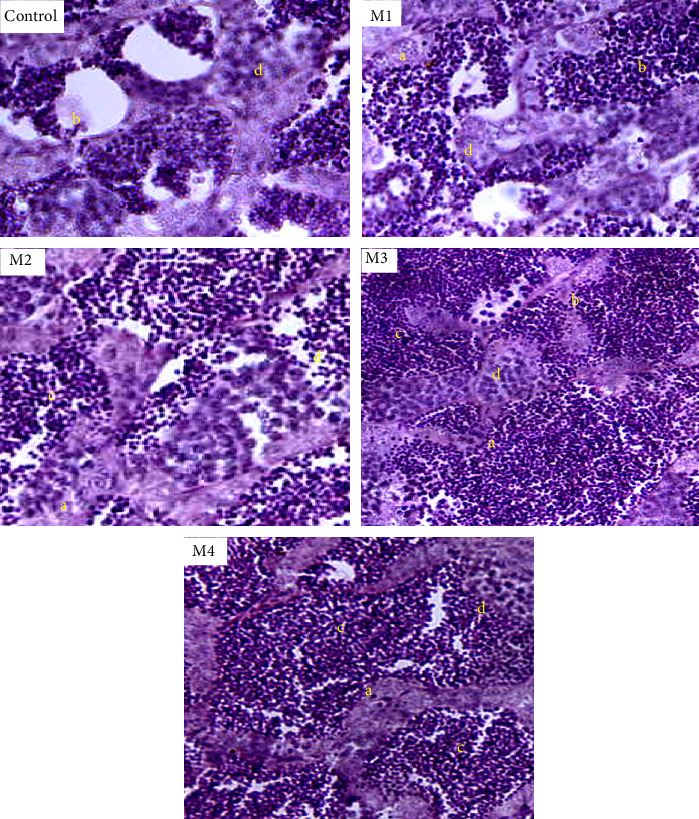
Histomicrographs showing the progression of testicular maturity in different groups with varying melatonin levels in the diets of *Devario aequipinnatus* during 60 days. Abbreviations used are a, spermatogonia; b, spermatocytes; c, spermatids; d, Sertoli cells. Arrows showing the testis undergoing active spermatogenesis and the visible spermatocytes (SC) and spermatozoa (SZ) in seminiferous tubules (ST) in M3 group.

In male fish, the preparatory phase of the reproductive cycle is marked by the initiation of gametogenesis, with stage I spermatids being the most advanced germ cells. During the prespawning phase, germ cell development progresses beyond stage I spermatids. The spawning phase is characterized by peak gametogenic activity, followed by a significant decline in testicular function during the postspawning phase. The various stages of testicular maturation are clearly depicted in Figure 6. In comparison to the control group, the M3 dose group (50 mg 100 g^−1^ feed) exhibited a significantly higher proportion of stage II spermatids and spermatozoa. This outcome is likely linked to enhanced spermatogenic activity, which is triggered by melatonin through the brain–pituitary–gonadal axis, increasing the sensitivity of Leydig cells to luteinizing hormone (LH). Aripin et al. [30, 35] reported that melatonin directly influences testosterone production in the testes by acting on Leydig cells. This aligns with the elevated testosterone levels observed in the melatonin-supplemented groups. The control and M1 groups (2 mg 100 g^−1^ feed) showed an abundance of spermatogonia and primary and secondary spermatocytes, indicating immature testicular development in these groups. Conversely, dietary melatonin doses above 50 mg 100 g^−1^, such as in the M4 group, resulted in a lower presence of spermatids compared to the M3 group. In the M3 group, the testicular lobules displayed stage II spermatids within ruptured thin lobule boundaries, indicative of advanced testicular maturity. The M3 group demonstrated a reproductive environment that was matured and ready for breeding, highlighting the dose-dependent effects of melatonin observed in this study. These findings are further supported by previous research by Aripin et al. [30, 35]. In conclusion, a melatonin supplementation dose of 50 mg 100 g^−1^ feed proved to be the most effective in promoting reproductive maturation in male fish.

### 4.4. Sperm Quantity and Quality

Male fish reproductive performance can be effectively assessed by evaluating sperm quantity (volume and concentration) and quality (motility, membrane stability and DNA integrity) [36, 37]. In this study, we examined sperm volume and concentration to understand the effects of exogenous melatonin, as shown in Figures 6 and 4. Melatonin-treated groups showed a significant increase in both sperm volume and concentration compared to the control group (*p*  < 0.05). The M3 group (50 mg 100 g^−1^ feed) recorded the highest sperm volume (16.4 ± 0.54 µm^3^) and concentration (4.36 ± 0.23 × 107 cell mL^−1^). These findings are consistent with a previous study by Aripin et al. [37, 38], which reported that melatonin treatment increased the number of mature spermatozoa in male catfish (*C. macrocephalus*). However, no significant changes in testicular maturity were observed in major carp (*C. catla*) following melatonin supplementation [5]. West et al. [38, 39] also highlighted melatonin's role in regulating spermatogenesis and reproductive maturation in fish. The improved sperm performance observed at the 50 mg 100 g^−1^ melatonin dose in our study suggests that exogenous melatonin positively influences fish reproductive maturity in a dose-dependent manner. While we did not specifically examine the antioxidant profile of sperm, it is plausible that dietary melatonin suppressed ROS accumulation, which might otherwise degrade sperm quality [39, 40].

### 4.5. Changes in the Levels of Sex Hormones

In fish, testosterone plays a critical role in spermatogenesis and has a significant impact on increasing sperm concentration, motility and duration [40, 41]. According to Langford et al. [41, 42], melatonin enhances the sensitivity of Leydig cells to gonadotropin hormone II (GTH-II), thereby boosting spermatogenic activity. Beyond its role in stimulating reproductive maturation via the brain–pituitary–gonadal axis, melatonin also acts directly on Leydig cells in the testes [42, 43]. GTH-II regulates testosterone production in Leydig cells, and the higher testosterone levels observed in the melatonin-treated groups likely contributed to the improved sperm scores. Testosterone also plays a protective role by preventing apoptosis in spermatocytes and spermatids, which further enhances sperm quality [43, 44]. In this study, sperm quality and quantity correlated with hormone levels across the treatments. The M3 group (50 mg 100 g^_1^ feed) recorded the highest sperm score (16.4 ± 1.11), while the control group had the lowest (4.37 ± 0.39) (*p*  < 0.05). Similarly, Rani et al. [44, 45] found that dietary melatonin administration in juvenile Asian sea bass significantly increased plasma testosterone levels. In contrast, previous studies reported a negative impact of exogenous melatonin on testosterone levels in masu salmon and *C. catla* [5, 45, 46]. Interestingly, there were no statistically significant differences (*p*  > 0.05) in hormone levels among female fish in this study. To further investigate reproductive performance, we analysed the variation of VG levels in the liver and muscle tissues of female fish. During vitellogenesis, VG, a protein synthesized in response to circulating oestrogens, is released into the bloodstream of maturing female fish [46, 47]. As a sex hormone indicator similar to testosterone, VG levels provide a comprehensive picture of reproductive health. With increasing melatonin supplementation, VG levels were significantly elevated (*p*  < 0.05) in both the liver and muscle tissues. The M3 group exhibited the highest levels, measuring 3.38 ± 0.22 nmol^−1^ in the liver and 1.83 ± 0.03 nmol^−1^ in the muscle. These findings highlight melatonin's central role in regulating testicular and ovarian physiology [47, 48]. Carnevali et al. [25, 26] also observed increased liver VG levels in zebrafish following melatonin exposure, which aligns with our results. Overall, the melatonin-supplemented groups showed significantly higher concentrations of sex hormones and VG, indicating early reproductive maturity in *D. aequipinnatus* in this study.

### 4.6. Effects on Fish Growth

In this study, dietary supplementation with melatonin significantly influenced the growth rates of *D. aequipinnatus* (Table 4). The SGR was significantly higher in the M3 group (3.95 ± 0.16, *p* < 0.05) than in the other groups, highlighting melatonin's growth-promoting effects at the 50 mg 100 g^−1^ supplementation level. The optimal melatonin dosage for *D. aequipinnatus* was identified as 63 mg/100 g of feed, based on broken-line regression analysis of WG, SGR and CF. This suggests that melatonin enhances growth performance in a dose-dependent manner. The growth-promoting effects of melatonin are likely linked to its role in regulating growth hormone production, as demonstrated in humans [23, 48]. Similarly, Singh et al. [25, 49] and Vera et al. [27, 50] reported that melatonin supplementation enhanced growth rates in tilapia (*O. niloticus*) and sharpsnout seabream (*Diplodus puntazzo*) up to an optimal level, beyond which growth rates declined. However, some studies have reported no significant relationship between melatonin supplementation and improved growth performance [8, 29, 51]. Consistent with the findings of Veras et al. [31, 52], it is possible that melatonin's external effects, such as stimulating locomotor activity and enhancing appetite, contributed to better growth outcomes in *D. aequipinnatus*. These findings emphasize the multifaceted role of melatonin in promoting growth, though its effects appear to be both dose- and species-dependent.

### 4.7. Immune and Antioxidant Enzyme Status

Melatonin's effects on fish immune systems have been shown to be circadian, with day length playing a particularly significant role [53].  Table 5 shows the results of our explanation of blood parameters (RBC, WBC, Hb and PCV) in order to gain insight into the immune system. The TEC and TLC were higher (*p*  < 0.05) in the M3 group (5.48 ± 0.13; 63.34 ± 0.01), while M4 showed a reduced level compared to the control group. The PCV was also higher in M3 (57.14 ± 1.34), while no significant change in levels was noticed between control with M1 and M2. The T4 group had the lowest level of TEC, TLC and PCV. Except for the M4 group, which showed elevated levels compared to the control, there was no statistically significant difference in Hb levels (*p*  < 0.05) across all melatonin groups. Improved growth rates are correlated with higher haematological scores, making them powerful tools for elucidating fish health. A sharp rise in TEC, TLC and PCV may help us understand the immune-stimulatory role of the provided melatonin, but this does not guarantee a concrete result given the absence of a set of immune parameters in this study. The M4 group (100 mg 100 g^−1^), however, showed decreased levels of blood parameters indicating health deterioration. High levels of melatonin have been linked to increased stress, which can be explained by the correlation between rising glucose and Hb levels [54].

Oocyte maturation and ovulation generate free radicals that have been linked to oxidative stress in fishes [51]. Oocyte quality was reportedly increased thanks to melatonin's ability to scavenge free radicals, which protects the ovary from damage [52]. SOD and another antioxidant enzyme called CAT are the body's main defences against free radicals. In contrast, MDA was a sensitive indicator of intracellular oxidative stress and enzymatic and nonenzymatic antioxidants in relation to the tempo of oocyte development [52]. We analysed the ovarian levels of the three most important antioxidant enzymes: SOD, CAT and MDA. As compared to the control group, all melatonin groups showed significant increases in SOD and CAT, while MDA levels decreased (*p*  < 0.05). T3 (50 mg 100 g^−1^) had the highest levels of SOD and CAT and the lowest levels of MDA among the groups. Consistent with our findings, Maitra and Hasan [52] found that melatonin levels increased in the ovary of *C. catla*, while the species was in its spawning or maturing phase, which they interpreted that melatonin played a role in lipid peroxidation. Similar scavenging effects of melatonin were also observed by Sentürk et al. [53] in rainbow trout, confirming the role of dietary melatonin in controlling oxidative stress in the ovary of *D. aequipinnatus*.

### 4.8. Effects on Glucose and Cortisol Level

Increased cortisol levels have been shown to influence gonad development, VG production, gamete quality and sex steroid levels in aquatic animals. This effect was independent of its impact on the GnRH and gonadotropin surge [54]. In this study, the supplemented groups had a significantly lower level of this hormone compared to the control group, suggesting that melatonin may have the potential to inhibit the subsequent increase in body cortisol level, thereby facilitating the clean adjustment of the fishes' reproductive performance. Consistent with previous research on sea bream and sea bass, we found that cortisol peaked at low melatonin levels (Figure 5a,b). Fish given 50 mg 100 g^−1^ melatonin had the lowest cortisol levels (323 ± 8.33 ng mL^−1^) of any of the groups. The lowest glucose level of 98.5 ± 2.21 ng mL^−1^ was also found in the same group as the lowest cortisol level. The increased need for glucose in the blood is a reflection of the increased energy expenditure associated with getting fully prepared for reproduction. We found, however, that optimal levels were maintained with control, supporting melatonin's beneficial function.

## 5. Conclusion

In conclusion, the exogenous melatonin had an effect on growth, reproduction, immunity and oxidative status. A melatonin supplementation dose of 63 mg 100^−1^ exerted better growth and reproductive performance of the fish compared to the other dietary levels tested. However, it was found that doses higher than this had a negative effect on these processes. The findings of the current study provide evidence for the enrichment of the reproductive cascade with melatonin, leading to better management of gametes. In light of these results, it is possible to simultaneously initiate a transfer of the recorded technical findings to rainbow farmers for the propagation of the endemic fishes of ornamental value. Studies of the expression patterns of various reproductive and growth genes through transcriptomic analysis should be the primary focus of future molecular-based research.

## Figures and Tables

**Table 1:** Ingredient composition of the experimental diets.

Ingredients	Experimental feeds (dry matter %)				
	Control	M1	M2	M3	M4
^a^Casein	55	55	55	55	55
^b^Corn starch	15	15	15	15	15
Cellulose	10	10	10	10	10
^c^Fish oil	8	8	8	8	8
^d^Vitamin–mineral mix	8	8	8	8	8
Guar gum	4	4	4	4	4
Total	100	100	100	100	100
Melatonin (mg 100 g^−1^)	0	2	10	50	100

^a^HiMedia, India.

^b^Local supplies.

^c^Cod liver oil from medical shop.

^d^Vitamin premix (mg/kg diet) contains vitamin A (16000 IU); vitamin B1 (17.80); vitamin B2 (48.00); vitamin B6 (29.52); vitamin B12 (0.24); vitamin C (800) as main constituent. Mineral premix (mg/kg diet): I (Ca (IO_3_)_2_) (1.63); Cu (CuSO_4_) (2.00); Fe (FeSO_4_) (21.10); Co (CoCl_2_) (0.24).

**Table 2:** Observed water quality parameters in dietary groups during the experimental period.

Parameters	Experimental groups				
	C	M1	M2	M3	M4
pH	6.8–8.2	6.9–8.1	6.8–8.05	7.04–7.9	7.2–8.09
Temperature (°C)	26.1–26.9	26.2–27	25.4–26.3	26.5–27.5	26.4–27.3
Dissolved oxygen (mg L^−1^)	5–7.2	5.1–7.31	5.04–7.2	5.21–7.09	5.3–7.4
Total hardness (mg L^−1^)	50.1–63.2	51.2–64.4	52.8–63.2	51.7–63.1	53.9–64.1
Total alkalinity (mg L^−1^)	60.2–75	61–79.3	63.3–78.7	61.8–78.1	62.4–77.6

**Table 3:** Hormonal levels of *Devario aequipinnatus* fed different levels of melatonin-incorporated diets.

Hormones	C	M1	M2	M3	M4
Testosterone (male) nmol^−1^	4.37 ± 0.398^a^	7.11 ± 0.514^b^	8.96 ± 0.493^b^	16.4 ± 1.11^c^	11.7 ± 0.358^d^
Testosterone (female) nmol^−1^	0.27 ± 0.05	0.3 ± 0.02	0.26 ± 0.04	0.23 ± 0.02	0.32 ± 0.03
Vitellogenin (liver) nmol^−1^	1.07 ± 0.09^a^	1.088 ± 0.1^a^	1.379 ± 0.26^b^	3.38 ± 0.22^d^	2.18 ± 0.24^c^
Vitellogenin (muscle) nmol^−1^	0.76 ± 0.03^a^	1.3 ± 0.09^b^	1.17 ± 0.058^b^	1.83 ± 0.03^c^	1.19 ± 0.05^b^

*Note:* Values are presented as means ± SE of three replicates. Different superscript letters (a, b, c, d) in each row show significant differences among treatments by Tukey's test (*p*  < 0.05).

**Table 4:** Biogrowth performance of *Devario aequipinnatus* fed varying levels of melatonin during the 60-day trial.

Parameters	Experimental groups				
	M1	M2	M3	M4	Control
Initial length (cm)	4.35 ± 0.45	4.35 ± 0.45	4.35 ± 0.45	4.35 ± 0.45	4.35 ± 0.45
Initial weight (g)	1.13 ± 0.15	1.13 ± 0.15	1.13 ± 0.15	1.13 ± 0.15	1.13 ± 0.15
Final length (cm)	5.94 ± 0.225^b^	6.35 ± 0.0291^c^	6.85 ± 0.05^d^	6.46 ± 0.0233^c^	5.17 ± 0.03^a^
Final weight (g)	1.89 ± 0.0567^a^	2.61 ± 0.246^b^	3.72 ± 0.185^c^	3.09 ± 0.265^b^	1.65 ± 0.0549^a^
Length increase (cm)	1.59 ± 0.2^b^	2 ± 0.02^c^	2.5 ± 0.05^d^	2.11 ± 0.02^c^	0.82 ± 0.03^a^
Weight gain (g)	0.763 ± 0.05^a^	1.48 ± 0.24^b^	2.58 ± 0.185^c^	1.96 ± 0.265^b^	0.523 ± 0.05^a^
SGR	1.71 ± 0.1^a^	2.75 ± 0.327^b^	3.95 ± 0.165^c^	3.32 ± 0.284^b,c^	1.26 ± 0.11^a^
Condition factor (CF)	1.02 ± 0.0	1.15 ± 001	1.16 ± 001	1.13 ± 0.01	0.92 ± 0.001

*Note:* Values are presented as means ± SE of three replicates. Different superscript letters (a, b, c, d) in each row show significant differences among treatments by Tukey's test (*p*  < 0.05).

Abbreviation: SGR, specific growth rate.

**Table 5:** Haematological observations in *Devario aequipinnatus* fed different levels of melatonin-incorporated diets.

Parameters	Experimental groups				
	C	M1	M2	M3	M4
TEC (million cells mm^−1^)	2.92 ± 0.10^b^	3.77 ± 0.09^b^	4.39 ± 0.15^c^	5.48 ± 0.13^d^	1.52 ± 0.13^a^
TLC (1000 cells mm^−1^)	5.42 ± 0.02^b^	5.45 ± 0.01^b^	5.94 ± 0.02^b^	6.34 ± 0.01^c^	4.28 ± 0.01^a^
PCV	45.66 ± 1.56^b^	48.125 ± 1.25^b^	45.30 ± 1.34^b^	57.14 ± 1.34^c^	43.78 ± 1.57^a^
Hb g dL^−1^	2.96 ± 0.12^a^	3.13 ± 0.08^a^	3.24 ± 0.04^a^	3.21 ± 0.15^a^	3.74 ± 0.06^a^

*Note:* Values are presented as means ± SE of three replicates. Different superscript letters (a, b, c, d) in each row show significant differences among treatments by Tukey's test (*p*  < 0.05).

Abbreviations: Hb, haemoglobin; PCV, packed cell volume; TEC, total erythrocyte count; TLC, leukocyte count.

**Table 6:** Status of antioxidant enzymes in the ovary of *Devario aequipinnatus* fed different levels of melatonin-incorporated diets.

Parameters	Control	M1	M2	M3	M4
SOD (U^−1^mL^−1^)	307 ± 3.64^a^	402 ± 7.52^b^	407 ± 6.45^b^	447 ± 8.33^c^	420 ± 3.81^c^
CAT Umin^−1^mg^−1^	20.31 ± 1.62^a^	34.87 ± 0.56^c^	26.26 ± 1.92^b^	37.64 ± 1.15^d^	32.53 ± 1.32^d^
MDA nmol mL^−1^	36.18 ± 1.5^d^	34.44 ± 0.73^c^	26.53 ± 0.74^b^	21.06 ± 1.3^a^	21.20 ± 1.37^a^

*Note:* Values are presented as means ± SE of three replicates. Different superscript letters (a, b, c, d) in each row show significant differences among treatments by Tukey's test (*p*  < 0.05).

Abbreviations: CAT, catalase; MDA, malondialdehyde; SOD, superoxide dismutase.

## Data Availability

The data supporting the findings of this study are available from the corresponding author upon reasonable request.
